# Biocompatible and Antimicrobial Electrospun Membranes Based on Nanocomposites of Chitosan/Poly (Vinyl Alcohol)/Graphene Oxide

**DOI:** 10.3390/ijms20122987

**Published:** 2019-06-19

**Authors:** Julián Andrés Tamayo Marín, Sebastián Ruiz Londoño, Johannes Delgado, Diana Paola Navia Porras, Mayra Eliana Valencia Zapata, José Herminsul Mina Hernandez, Carlos Humberto Valencia, Carlos David Grande Tovar

**Affiliations:** 1 Escuela de Ingeniería de Materiales, Facultad de Ingeniería, Universidad del Valle, Calle 13 No. 100-00, Santiago de Cali 760032, Colombia; julian.tamayo@correounivalle.edu.co (J.A.T.M.); sebastian.ruiz.londono@correounivalle.edu.co (S.R.L.); valencia.mayra@correounivalle.edu.co (M.E.V.Z.); jose.mina@correounivalle.edu.co (J.H.M.H.); 2 Grupo de Investigación Biotecnología, Facultad de Ingeniería, Universidad de San Buenaventura Cali, Carrera 122 No. 6-65, Cali 76001, Colombia; jdelgado1@usbcali.edu.co (J.D.); dpnavia@usbcali.edu.co (D.P.N.P.); 3 Escuela de Odontología, Grupo biomateriales dentales, Universidad del Valle, Calle 13 No. 100-00, Cali 76001, Colombia; carlos.humberto.valencia@correounivalle.edu.co; 4 Programa de Química, Facultad de Ciencias, Universidad del Atlántico, Carrera 30 Número 8-49, Puerto Colombia 081008, Colombia

**Keywords:** antibacterial nanofibrous membranes, chitosan, electrospinning, graphene oxide, polyvinyl alcohol

## Abstract

Tissue engineering is gaining attention rapidly to replace and repair defective tissues in the human body after illnesses and accidents in different organs. Electrospun nanofiber scaffolds have emerged as a potential alternative for cell regeneration and organ replacement. In this paper, porous membranes, based on nanofibrous chitosan (CS), polyvinyl alcohol (PVA), and graphene oxide (GO), were obtained via electrospinning methodology. Three different formulations were obtained varying GO content, being characterized by Fourier Transform Infrared spectroscopy (FTIR), scanning electron microscopy (SEM), and energy dispersive spectroscopy (EDS). In vitro tests were carried out, consisting of hydrolytic degradation inside simulated biological fluid (SBF), and in vivo tests were carried out, where the material was implanted in Wistar rats’ subcutaneous tissue to determine its biocompatibility. The antibacterial activity was tested against Gram-positive bacteria *Bacillus* *cereus* and *Staphylococcus* *aureus,* and against Gram-negative *Salmonella* *enterica* and *Escherichia* *coli*, by contact of the electrospun nanofiber scaffolds above inoculum bacterial in Müeller Hinton agar with good inhibition only for scaffolds with the higher GO content (1.0%). The results confirmed good biocompatibility of the nanofibrous scaffolds after in vivo tests in Wistar rats, which evidences its high potential in applications of tissue regeneration.

## 1. Introduction

Currently, in the world, millions of people are affected by bone defects due to accidents, traumas, tumors, natural aging, bone fractures, obesity, and physical activity [1]. Due to this problem, the search for biomaterials with application in tissue engineering for the development of three-dimensional porous structural materials that imitate bone behavior has increased. This porous framework must possess the properties of a natural bone, such as biocompatibility, biodegradability, support for cell adhesion, proliferation, and cell growth, to overcome accidents, traumas, tumors, natural aging, bone fractures, obesity, and physical activity [1]. The primary function of the scaffolding is to mimic the natural extracellular matrix temporarily, while the new bone is growing, which serves as a biological substitute for adhesion, allows cell migration, and, finally, guides the development of the new tissue with the appropriate functions [2].

Typically, various materials have been used for the manufacture of scaffolds, especially of the polymeric type, due to its excellent biocompatibility, adjustable chemical composition, suitable biological rearrangement, and acceptable degradation. However, the main drawback of natural polymers is their low mechanical resistance, which makes them unsuitable for a bone scaffold [3]. For this reason, it is necessary to reinforce the mechanical and thermal properties of the materials used as scaffolds with nanocomposites, which can increase mechanical and thermal resistance, as well as the barrier and antimicrobial properties. Therefore, the development of nanocomposites based on biopolymers, such as chitosan (CS), thanks to its biocompatibility, biodegradability, physiological inertness, remarkable affinity to proteins, and antibacterial, hemostatic, fungistatic, and antitumor properties, becomes an excellent alternative for the design of biomaterials for cell regeneration [4].

Graphene is a solid material composed of a thin single atomic sheet of sp2 carbon atoms [5,6,7]. Functionalization of graphene for several uses in biomedicine (e.g., biosensors, nanocarriers, and devices for cell imaging and phototherapy for cancer) is an active area nowadays [8,9,10,11,12]. Usually, it is synthesized from the allotropic form graphite, one of the most abundant chemical elements in nature [13]. Several nanocomposites based on graphene and other materials have been used for stimulating neural stem cell adhesion, proliferation, differentiation, and neural regeneration [14].

Graphene oxide (GO), one of graphene’s derivatives, has become a highly used nanofiller thanks to its high mechanical resistance, chemical stability, large surface area, and low toxicity [15]. Also, due to its antibacterial properties and a high surface/volume ratio, GO is a promising material for the development of antimicrobial surfaces [16]. Graphene oxide nanocomposites have been prepared to improve surface roughness and promote neural growth [14,17,18,19,20].

Recently, our group prepared antimicrobial films based on nanocomposites of chitosan/polyvinyl alcohol/graphene oxide by the drop-casting method, which showed excellent biocompatibility in the subcutaneous tissue of Wistar rats, antimicrobial activity against Gram-positive and Gram-negative bacteria, and better thermal and mechanical stability with the increase of graphene oxide [21]. We also prepared scaffolds by the freeze-drying method based on CS–GO nanocomposites demonstrating excellent biocompatibility after 30 days of implantation in Wistar rats’ subcutaneous tissue. Reabsorption of the material by phagocytic activity and new bone formation in experiments on critical size defects has also been demonstrated [22,23]. However, there is a growing need to obtain materials with a tunable size of porosity to better stimulate cell adhesion and regeneration through the use of these devices.

Micro/nanofibrous scaffolds have been investigated extensively for tissue engineering and drug delivery [24]. Since micro/nanofibrous scaffolds mimic the natural extracellular matrix (ECM), they stimulate cell adhesion, proliferation, migration, and differentiation better than particulate structures. By the other side, due to its higher surface-area to volume ratio and higher interconnected porosity, cell adhesion and proliferation occurs quickly, which makes it a promising material for tissue engineering [25].

Electrospinning technique has been used to develop these controlled nanostructures with high porosity ratio for the formation of polymer fibers. This technique has advantages because, by using electric forces, fibers with diameters ranging from 2 nm to several micrometers can be obtained using solutions of both natural and synthetic polymers [26]. This process offers unique capabilities for the production of new nanofibers and natural tissues with controllable pore structure [27]. Electrospun nanofibrous membranes can be easily tuned to match irregular bone defects to promote osteointegration [28]. For all of the above reasons, electrospinning of polymeric micro/nanofibrous scaffolds has the potential for application in traumatic or disease states, such as in skin regeneration or the treatment of cancer [29].

Even though several studies on CS/ polyvinyl alcohol (PVA)/GO films and electrospun polymeric micro/nanofibrous scaffolds have been investigated extensively in the literature, there is a lack of information about the physical, chemical, and mechanical characterization of the nanofibrous membranes, and their biocompatibility and antimicrobial performance. Therefore, this research proposed the study of biodegradable nanofibrous membranes based on PVA/CS/GO with potential applications for tissue regeneration and antimicrobial devices.

## 2. Results and Discussion

### 2.1. GO Synthesis and Characterization

GO synthesis followed the methodology used by Mangadlao [30]. GO synthesis and characterization were previously reported by our group [21].

### 2.2. Electrospun CS/PVA/GO Composite Nanofibrous Membranes Characterization

#### 2.2.1. Fourier-Transform Infrared Spectroscopy (FTIR)

**Figure 1:**
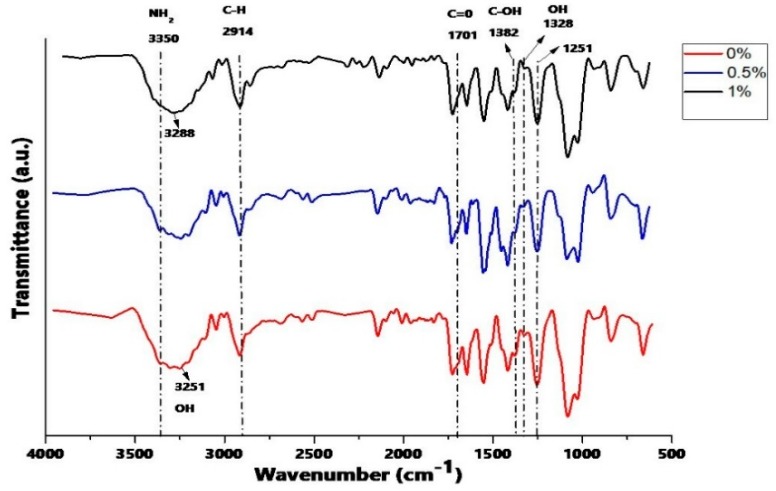
Attenuated total reflectance Fourier-transform infrared spectroscopy (ATR-FTIR) of electrospun chitosan (CS)/polyvinyl alcohol (PVA)/graphene oxide (GO) composite nanofibrous membranes with different GO amounts (0%, 0.5%, and 1.0%).

In Figure 1, the ATR-FTIR spectrum of the scaffolds are shown. It was observed that a band appears at 1701 cm^−1^ due to the stretching vibration of carbonyl groups C=O (-COOH) at the edges of GO. The vibration band coupled in the OH plane (1328 cm^−1^) becomes more pronounced due to the destruction of the original hydrogen bonds of CS/PVA compounds and the formation of a strong interaction between CS, PVA, and GO, with the increase in the amount of GO. The C–OH bond observed at 1382 cm^−1^ is weakened with the increase in GO due to the strong hydrogen bond. On the other hand, no changes were noted in the functional groups in the compound system, although it is known that generally between the CS and the GO there will be hydrogen bonds that stabilize their compounds. The bands of the secondary amino groups and amide groups formed would be overlapping with the bands of amino groups and amide groups of the CS [31]. Furthermore, the band at 3251 cm^−1^, due to the stretching vibration of the OH group, was displaced to higher wavenumbers (3288 cm^−1^) and was further expanded, which can be attributed to the hydrogen interaction of GO with the mixture CS-PVA [32].

#### 2.2.2. Scanning Electron Microscopy (SEM)

The morphology of the electrospun fibers is controlled by various parameters, such as the applied voltage, the flow velocity of the solution, the distance between the nozzle and the collector, and, especially, the concentration and surface tension of the solution [33].

**Figure 2:**
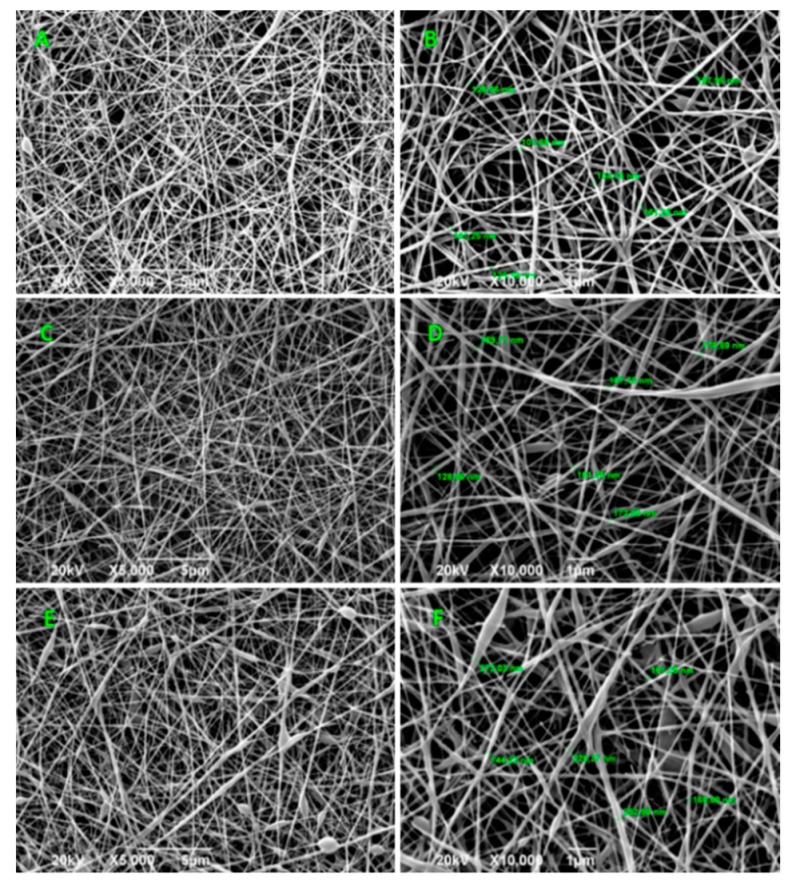
SEM images of electrospun CS/PVA/GO composite nanofibrous membranes with different GO amounts (0%, 0.5%, and 1.0%). Images (**A**,**C**,**E**) at 500×, and images (**B**,**D**,**F**) at 10,000×. For all the experiments, the voltage used was 20 kV.

As the Figure 2 shows, the composite nanofibers exhibit a random fibrous morphology and an interconnected porous structure, where the effect of the addition of GO is reflected in the increase of the average diameters of the fibers, due to the rise in the viscosity of the mixture with the addition of the GO [34,35]. The diameter of the fibers increased in average 144.45, 152.94, and 202.79 nm for GO percentages of 0%, 0.5%, and 1% by weight, respectively (Table S1).

Viscosity refers to the resistance exerted by a fluid against a tangential deformation. This resistance to flow is generated from the friction between the molecules. The increase in the viscosity can be justified because, when increasing the concentration of GO, the quantity of electrospun CS/PVA/GO composite nanofibrous membranes that interacted with each other, both with the polymeric molecules, increased. In addition, the formation of agglomerates in suspension was induced and they act as a resistance to the flow. When the jet is generated, the solution exerts strength to being stretched by the electric field, creating larger diameter fibers [36,37]. However, the defects presented can be attributed to an agglomeration of the GO sheets due to the high concentration that was used. On the other hand, it can be attributed to the effect of the residual solvent that was not evaporated during the process. Therefore, the flat fibrillar structure was not obtained in some regions of the material [36,38].

#### 2.2.3. Degradation in a Simulated Biological Fluid (SBF)

**Figure 3:**
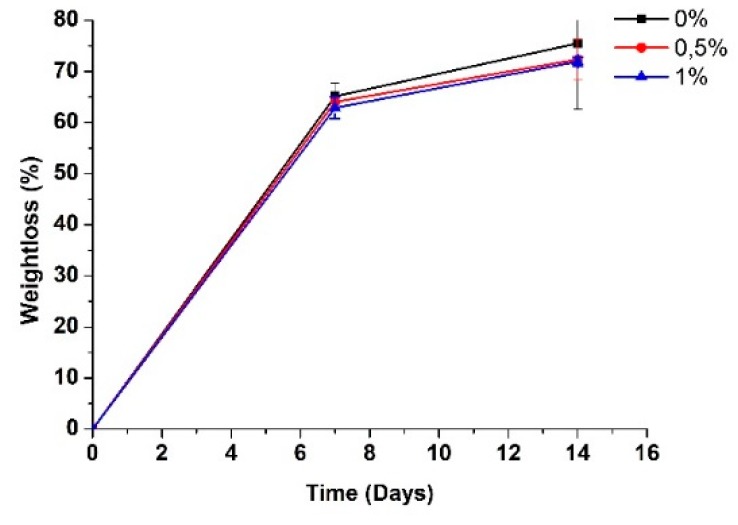
Weight loss of electrospun CS/PVA/GO composite nanofibrous membranes with different GO amounts (0%, 0.5%, and 1.0%), after several periods of immersion in a simulated biological fluid SBF.

Figure 3 shows the results of the degradation percentage (weight loss) of the scaffolds with different proportions of GO after 14 days of being subjected to the process of hydrolytic degradation in a simulated biological fluid (SBF). The rate (%) of weight loss was calculated by employing Equation (1). It can be observed that the incorporation of GO in the polymer matrix improved the stability of the scaffolds against the SBF. The weight loss was lower for the samples with higher GO content, the samples with 0% GO presented a final degradation of 75.4%, which is higher in comparison with the samples that contained some percentage of GO: the scaffolds with 0.5% GO exhibited an ultimate degradation of 72.33% and the frameworks with 1% GO showed the lowest deterioration of 71.90%. Therefore, it was observed that with a higher content of GO there is a lower weight loss, attributed to the higher content of GO in the CS/PVA binary mixture due to hydrogen bonds with the CS. Since many secondary bonds are present, these forces will multiply, and it will have high energy along with these links, evidenced in more excellent stability in the polymer chains [39].

The degradation behavior is explained by the interaction of the SBF with the CS and PVA. This interaction generates random excisions of the polymer chains since the introduction of water inside the polymer matrix causes the hydration of the molecules, rupture of hydrogen bonds, swelling, and, finally, the hydrolysis of unstable bonds [40]. Ultimately, resulting in a decrease in molecular weight and, therefore, higher susceptibility to a weight loss of the samples [41].

(1) $$W_{l}\;\left(\%\right)=\frac{W_{0}-W_{d}}{W_{0}}\;\times 100$$

**Figure 4:**
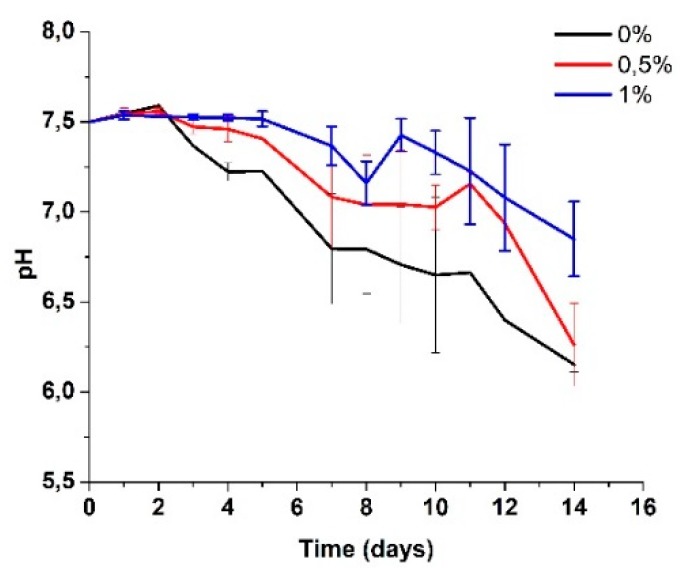
Change in pH in the SBF after several days of immersion of the electrospun CS/PVA/GO composite nanofibrous membranes with different GO amounts (0%, 0.5%, and 1.0%).

In Figure 4, the average variation in pH of the SBF solution is shown. The drop in pH has been associated with the attack on amorphous zones because they retain more acidic species and because their blocks are more susceptible to degradation since they do not have segments of crystalline zones that hold them together, according to Figueira Maldonado [42]. Another possible cause could be the remaining acetic acid present in the scaffolds due to the production process used and the degradation of byproducts typical in CS, such as glucosamine or N-acetylglucosamine, which can also be found in the extracellular tissue matrix of humans, making them harmless when released into the human body [43].

The pH of the biological media is a fundamental constant for the maintenance of vital processes. The enzymatic action and the chemical transformations of the cells are carried out within strict pH ranges. In the human body, the admissible scales that promote life and maintenance of vital functions oscillate between 6 and 8, as determined by the ASTM F1635 standard and confirmed in various investigations [44,45], which indicates that the values obtained during the degradation of the scaffolds are within the known ranges for biological processes.

#### 2.2.4. SEM of the Electrospun CS/PVA/GO Composite Nanofibrous Membranes after Immersion in SBF

**Figure 5:**
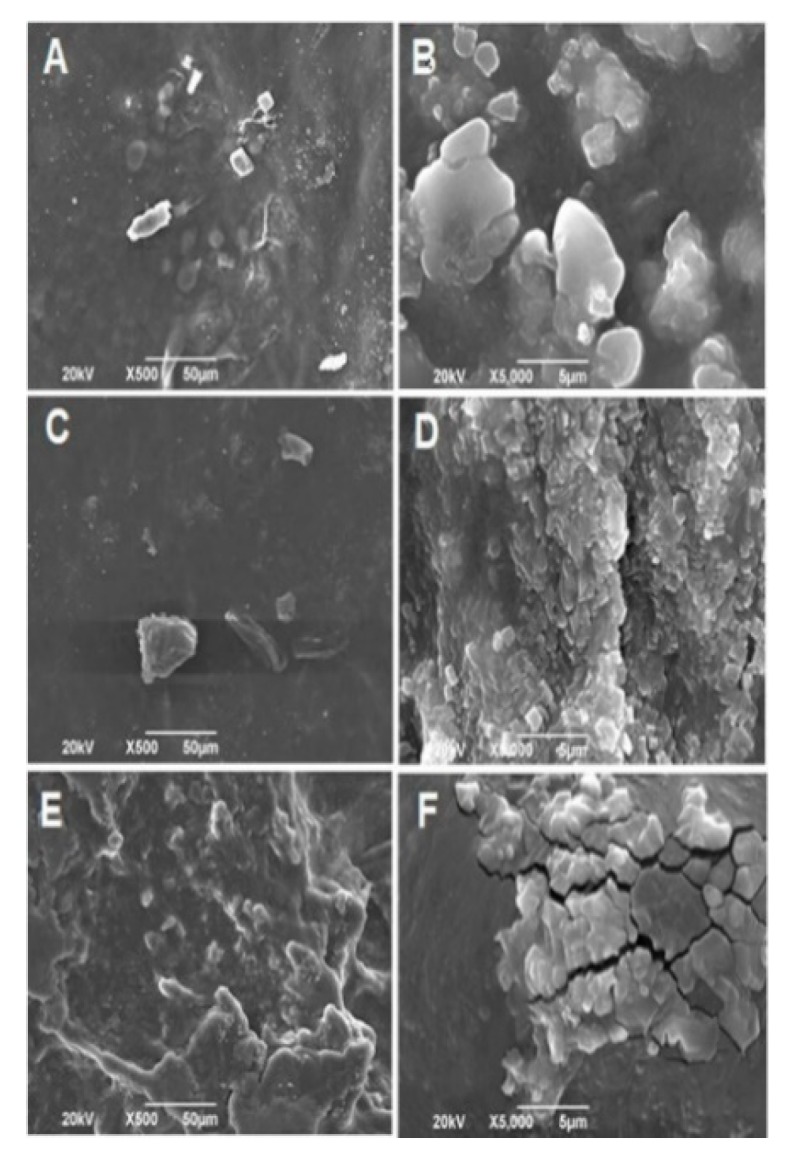
SEM images of the electrospun CS/PVA/GO composite nanofibrous membranes after fourteen days in the degradation process in SBF. (**A**,**B**) 0% GO, (**C**,**D**) 0.5% GO, and (**E**,**F**) 1.0 % GO. Images (**A**,**C**,**E**) at 500×, and images (**B**,**D**,**F**) at 10,000×. For all the experiments, the voltage used was 20 kV.

It was expected that the characteristic fibrillar structure of these materials would resist dissolution during the incubation in SBF. However, it could be seen by the SEM that there are notable differences between the morphologies before and after the degradation process in SBF (Figure 2 and Figure 5), respectively, giving evidence of a rough surface and with fibers fused together. The fibrillar and porous structure disappeared, possibly due to the swelling of the fibers, which was attributed to the hydrophilic character of CS and PVA in a high degree of hydrolysis and medium molecular weight [44].

The immersion of the scaffolds in SBF was also carried out to evaluate the capacity of apatite formation on the surface of the material. The SEM images showed that, in some places, the scaffolds were covered by hydroxyapatite (HA) crystals, as evidenced for the scaffolding with 0.5% GO (Figure 5D) after 14 days of immersion, confirming the capacity of the material to stimulate the formation of new bone. This was verified through dispersive energy spectroscopy (EDS, data not shown) where it showed that the peak signals of Ca and P were present in the scaffolds, with proportions of calcium of 7.09% and phosphorus of 3.11%, which confirms the formation of apatite. In addition to these main elements, the presence of small amounts of Na, Cl, K, and Mg was detected. The above components were derived from SBF.

Figure 5 shows the SEM images of the surface of the CS/PVA/GO scaffolds that were successfully made by the electrospinning method, showing a fibrillar morphology, as expected. With a volume ratio of PVA/CS of 7:3 and different concentrations of GO (0%, 0.5%, and 1% by weight of CS) the nanofibers were uniform, smooth, continuous and without defects in almost the entire sample.

The results showed the formation of hydroxyapatite compounds. Therefore, CS/PVA/GO scaffolds could provide a promising construction for the application of bone tissue engineering, as long as the chemical stability in SBF is improved. However, it is advisable to perform tests in subdermal implantation in the longer term to verify if there is bone ectopic formation or to perform tests on critical size intraosseous defects to study the osteogenic capacity of the biomaterial.

#### 2.2.5. Antibacterial Activity

As shown in Table 1, no inhibition of pathogens was observed in the treatments with 0% and 0.5% of GO. Although inhibition against Gram-positive and Gram-negative bacteria can occur due to GO and chitosan [46,47], the fact that there was no inhibition when there is only chitosan (0% GO) shows the strong interaction of hydrogen bonds between chitosan and PVA, which decreases the solubility of chitosan in the medium [45]. This interaction prevents its interaction with the cell membrane of the microorganisms, causing no inhibition to occur.

This explanation suggests that the inhibition found in the treatment with 1.0% GO is mainly due to the GO effect. The sharp atomic edges of the GO penetrate the cell’s membrane and destabilize its integrity. Chemically, they can promote lipid peroxidation induced by the natural oxidative nature of GO depending directly on their concentration in the medium [46,47]. This inhibition was presented against all the bacteria evaluated, which agrees with that found by other authors against Gram-positive bacteria [48,49] and Gram-negative bacteria [48,49,50,51,52,53,54,55].

#### 2.2.6. Biomodel Tests In Vivo

**Figure 6:**
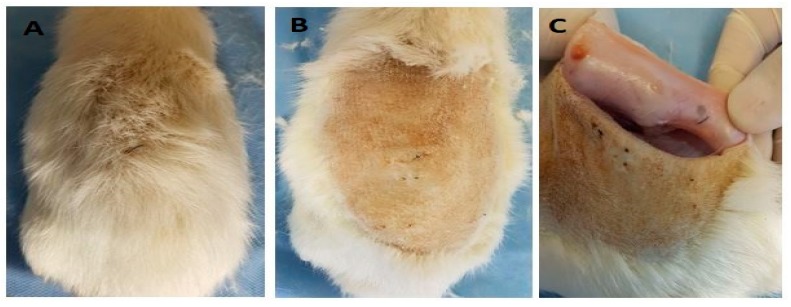
The dorsal area of the rat Wistar after 30 days of the implantation: (**A**) hair recovery, (**B**) absence of injuries and infections, and (**C**) internal surface of the skin where the implanted samples are encapsulated by scar tissue.

After 30 days of implantation in the biomodels, the samples were recovered. In all cases, repair of the created surgical defect was observed. Furthermore, all the biomodels showed a hair recovery and absence of injuries and infections in the intervened areas with healthy healing and restoration of the tissue architecture (Figure 6). The material, although quite compatible, generated an inflammatory response to a foreign body where the cells surrounded the fragments with a fibrous capsule and the rest of the soft tissues with healthy appearance, for the case of higher GO content (1.0%).

**Figure 7:**
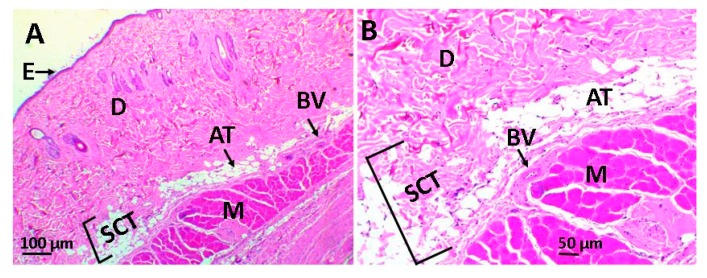
Image of the control sample using the hematoxylin and eosin technique. Image (**A**) at 4× and image (**B**) at 10×. E: epidermis, D: dermis, AT: adipose tissue, BV: blood vessel, SCT: subcutaneous cellular tissue, and M: muscle.

Figure 7 shows the images of the histological study of the subcutaneous tissue after 30 days of implanting the scaffolds in the biomodels (Wistar rats). In Figure 7, we have the histological image of the control sample (porcine collagen), where it was observed that there was resorption of the material with the recovery of the anatomical architecture of the tissue.

**Figure 8:**
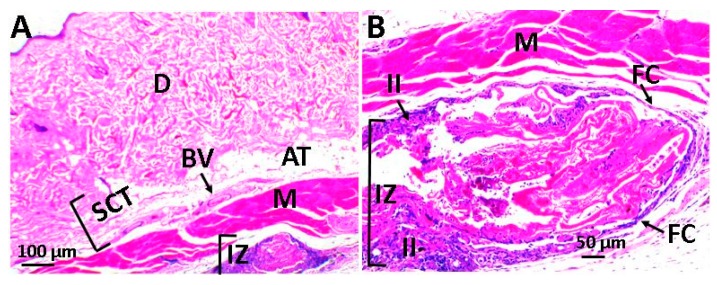
Scaffold with 0% GO using the hematoxylin and eosin technique. Image (**A**) at 4× and image (**B**) at 10×. D: dermis, AT: adipose tissue, BV: blood vessel, SCT: subcutaneous cellular tissue, II: inflammatory infiltrate, IZ: implantation area, M: muscle, and FC: fibrous capsule.

Figure 8 corresponds to scaffolds with 0% GO, recovery of tissue architecture, and fragments of the material in the process of resorption with the presence of inflammatory infiltrate are noted. It was also possible to observe the presence of a fibrous capsule (FC), corresponding to a series of collagen fibers that surrounded the biomaterial and that is a common finding in the experiments that include the implantation of materials, as part of the healing process within the chronic inflammation phase.

**Figure 9:**
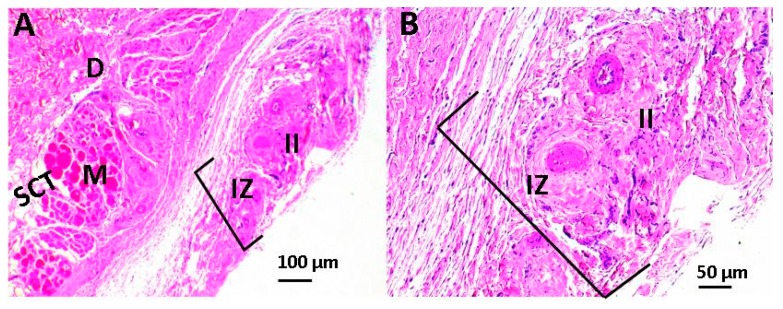
Scaffold with 0.5% GO using the hematoxylin and eosin technique. Image (**A**) at 4× and image (**B**) at 10×. D: dermis, AT: adipose tissue, SCT: subcutaneous cellular tissue, II: inflammatory infiltrate, IZ: implantation area, and M: Muscle.

**Figure 10:**
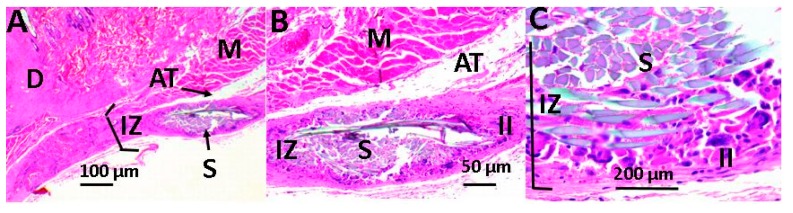
Scaffold with 1% GO using the hematoxylin and eosin technique. Image (**A**) at 4× and image (**B**) at 10×, Image (**C**) at 40×. D: dermis, AT: adipose tissue, II: inflammatory infiltrate, S: scaffold, IZ: implantation area, and M: muscle.

Figure 9 and Figure 10 correspond to the implanted material with graphene oxide content. In both of them, the recovery of tissue architecture is observed. Figure 9 corresponds to the material with 0.5% GO, which shows a normal healing process, with the presence of traces of material in the process of resorption and are surrounded by fibrous tissue with the presence of inflammatory cells, although the inflammatory response seems to be lower in scaffolds with 0.5% GO. In the case of structures with 1% GO (Figure 10), a continuity solution was observed in the tissue, healing was delayed, and a greater inflammatory process was apparent when compared to the two previous cases.

In all cases, a normal healing process was observed with the recovery of the tissue architecture. In scaffolds with 0.5% graphene oxide, traces of the material being phagocytized by an inflammatory infiltrate were observed, whereas in frameworks with 1% graphene oxide, resorption was minimal after 30 days of implantation and the situation has been solved as a reaction to a foreign body that has encapsulated the scaffold (FC).

## 3. Materials and Methods

### 3.1. Materials

For graphene oxide synthesis, graphite flakes (99.8%) were used (Alfa Aesar, Tewksbury, MA, USA). Concentrated sulfuric acid (H_2_SO_4_), potassium permanganate (KMnO_4_), hydrogen peroxide (H_2_O_2_), and isopropanol were supplied by Merck (Burlington, MA, USA). For the production of the films, chitosan of low molecular weight (Mv 144.000 g/mol) and a deacetylation degree between 89–90%, polyvinyl alcohol (PVA), with hydrolysis between 87–89% and viscous molecular weight of 93,000 g/mol was used (Sigma-Aldrich, Palo Alto, CA, USA). Glacial acetic acid comes from Merck (Burlington, MA, USA). For the elaboration of the simulated biological fluid, NaCl, K_2_HPO_4_ 3H_2_O, CaCl_2_, Na_2_SO_4_, and tris-(hydroxymethyl aminomethane) [(CH_2_OH)_3_CNH_2_] were acquired from Sigma Aldrich (Palo Alto, CA, USA); NaHCO_3_, KCl, and MgCl_2_ 6H_2_O from Fisher Chemical (Pittsburgh, PA, USA); and hydrochloric acid (HCl) from Merck (Burlington, MA, USA). All reagents used were analytical degree.

### 3.2. Methods

#### 3.2.1. GO Synthesis

GO synthesis followed the methodology used by Mangadlao [30]. GO synthesis and characterization were previously reported by our group [21].

#### 3.2.2. Preparation of Electrospun CS/PVA/GO Composite Nanofibrous Membranes

Scaffolds were manufactured by the electrospinning method using a vertical configuration. A high voltage source (Gamma High Voltage Research INC., Model E30) was used. It was connected to the nozzle and the collector, then the polymer solution was introduced in a 10 mL syringe, and, by means of a polypropylene hose, it was coupled to the electrospinning nozzle in stainless steel. Finally, a voltage of 22 kV was applied and a pump (Braintree Scientific INC) was used to control the flow of the solution, maintaining it at 1.0 mL/h. As a result of the positive polarization of the solution and the electric field, the solution was attracted by the negatively charged collector, thus forming the fibers, which were collected on a slotted Teflon mold, which is arranged on an aluminum sheet. The whole process was carried out at a temperature (21–30 °C) and relative humidity (45–60%), average to the environmental conditions of the city of Santiago de Cali, Colombia.

The solution for electrospinning was carried out with individual solutions of CS at 5% (*w*/*v*) dissolved in 2% acetic acid (*v*/*v*), 8% PVA solution (*w*/*v*) dissolved in distilled water at 80 °C, and 300 rpm. The GO was dispersed in the ultrasonic medium. A volume ratio of PVA:CS of 7:3 was used, and the GO was added concerning 1% by weight of the CS.

### 3.3. Characterization

#### 3.3.1. Fourier Transform Infrared Spectroscopy (FTIR)

The chemical identification of the films was carried out using FTIR in the ATR mode (attenuated total reflectance) (Shimadzu, Kyoto, Japan).

#### 3.3.2. Scanning Electron Microscopy (SEM)

The morphological inspection of the surfaces of the film was carried out through a scanning electron microscope (SEM) (JEOL JSM-6490LA, Musashino, Tokyo, Japan). The working conditions were 20 kV and mode of secondary backscattered electrons. All the samples were coated with gold to create an electronic density in the material since the polymers lack it.

#### 3.3.3. Degradation in Simulated Biological Fluid

The hydrolytic degradation was carried out following the procedure outlined in the American Society for Testing and Materials (ASTM) F1635-16 standard. The scaffolds were immersed in a simulated biological fluid (SBF) at 37 °C for 14 days in a Memmert IN 110 incubator (Memmert, Schwabach, Germany). The SBF was prepared according to the method proposed by Kokubo and Takadama [56], and the degradation was evaluated by examining the weight of the films before and after immersion for different periods (1, 3, 5, 7, and 14 d).

The initial weight of the samples before immersion was recorded as $W_{0}$ and the weight after drying for 48 h in the incubator at 37 °C was recorded as $W_{d}$. The weight loss (% $W_{l}$) was calculated according to Equation (1).

Each sample was immersed in 15 mL of SBF, and, in each period, three samples were evaluated per formulation. The pH of the SBF was measured every day until the total test time was completed using an Accumet AB150 pH meter (Fisherbrand, Ottawa, ON, Canada). The morphology of the films after drying was studied using SEM.

#### 3.3.4. Antimicrobial of Electrospun CS/PVA/GO Composite Nanofibrous Membranes Assay

The antimicrobial activity of the electrospun CS/PVA/GO composite nanofibrous membranes was evaluated against Gram-positive bacteria, Bacillus cereus (ATCC 13061) and Staphylococcus aureus (ATCC 55804), and Gram-negative bacteria, Salmonella enterica (ATCC 13311) and Escherichia coli (ATCC 11775), by contact of the electrospun CS/PVA/GO composite nanofibrous membranes above inoculum bacterial in agar. The methodology used by Ruiz et al. (2018) was followed without modifications [21]. The test was repeated three times for each of the treatments.

#### 3.3.5. Biomodels Test In Vivo

The inflammatory response to the implantation of films in the subcutaneous tissue was measured. Samples of CS–PVA films with different percentages of GO (three replicates per formulation) with 10 mm in diameter and 2 mm in thickness were implanted in the subdermal tissue of three adult Wistar rats, in preparations made on the dorsal surface, according to the recommendation of ISO 10993-6. As a control sample, commercial porcine collagen with the same dimensions was used. All the biomodels were supplied by the Bioterium of the Faculty of Medical Sciences of the Universidad del Valle. The procedures carried out were approved by the Animal Ethics Committee of the Universidad del Valle, by the CEAS 001-016 certificate of May 20th of 2016.

After 30 days of implantation, the samples were recovered, fixed in buffered formalin, dehydrated in alcohol solutions of ascending concentration (70%, 80%, 95%, and 100%), diaphanized with xylol and infiltrated with paraffin for later cutting to 4 µm. The samples were processed for histological analysis by hematoxylin and eosin (H&E) and masson trichromacy (MT) techniques.

## 4. Conclusions

In this research, we demonstrated a simple protocol for obtaining electrospun scaffolds based on CS/PVA/GO nanocomposites, which showed adequate chemical and biological properties for their application in tissue engineering. The addition of GO in the electrospun scaffolds did not interfere with the correct formation of the fibrillar structure. Rather, it gave a more excellent stability against the degradation in SBF. It was also evidenced through EDS that in some places the scaffolds were covered by a mild apatite layer.

Finally, the material that presented the best tissue biocompatibility was the one that had a GO content of 0.5%. Interestingly, there was a better antibacterial response when the GO was increased to 1%, being active both for Gram-positive bacteria and Gram-negative bacteria. However, the inflammatory response also increased and the degradability was reduced when it was implanted. According to this observation, the use of biomaterial the most suitable in subdermal applications is the scaffold with GO at 0.5%, and scaffolds with 1% GO should be recommended in areas where an antibacterial effect is needed, such as in wounds exposed and infected skin, since the inflammatory response seems to be higher as the percentage increases. It is also necessary to perform additional studies to evaluate the osteogenic effect in applications for bone tissue engineering.

## Figures and Tables

**Table 1:** Inhibition of electrospun CS/PVA/GO composite nanofibrous membranes against bacterial strains.

Strain	0% GO	0.5% GO	1.0% GO
*Bacillus cereus*	---	--	+
*Staphylococcus aureus*	---	--	+
*Salmonella spp*	---	--	+
*Escherichia coli*	---	--	+

(+) Weak inhibition of the pathogen; (--) Weak pathogen growth; (---) Complete growth of the pathogen.

## Supplementary Materials

Supplementary material Table S1 can be found at https://www.mdpi.com/1422-0067/20/12/2987/s1.
