# Supplementary material for: Biocompatible and Antimicrobial Electrospun Membranes Based on Nanocomposites of Chitosan/Poly (Vinyl Alcohol)/Graphene Oxide
Source: Int J Mol Sci. 2019 Jun 19;20(12):2987. doi: 10.3390/ijms20122987 (PMC6627348; doi:10.3390/ijms20122987)
Supplement: Supplementary file 1 [file ijms-20-02987-s001.pdf]

Supplementary Materials: Biocompatible and antimicrobial electrospun membranes based on nanocomposites of chitosan/poly (vinyl alcohol)/graphene oxide

Julián Andrés Tamayo[1], Sebastián Ruiz[1], Johannes Delgado[2], Diana P. Navia[2], Mayra Eliana Valencia Zapata[1], José Herminsul Mina Hernandez[1], Carlos Humberto Valencia[3] and Carlos David Grande Tovar[4*]

[1] Escuela de Ingeniería de Materiales, Facultad de Ingeniería, Universidad del Valle, Calle 13 No. 100-00, Santiago de Cali 760032, Colombia; sebastian.ruiz.londono@correounivalle.edu.co (S.L.); julian.tamayo@correounivalle.edu.co (J.T.); valencia.mayra@correounivalle.edu.co (M.V.); jose.mina@correounivalle.edu.co (J.M.)

[2] Grupo de Investigación Biotecnología, Facultad de Ingeniería, Universidad de San Buenaventura Cali, Carrera 122 # 6-65, 76001 Cali, Colombia; jdelgado1@usbcali.edu.co (J.D.); dpnavia@usbcali.edu.co (D.N.)

[3] Escuela de Odontología, Grupo biomateriales dentales, Universidad del Valle, Calle 13 No. 100-00, 76001 Cali, Colombia; carlos.humberto.valencia@correounivalle.edu.co

[4] Programa de Química, Facultad de Ciencias, Universidad del Atlántico, Carrera 30 Número 8-49, Puerto Colombia 081008, Colombia

* Correspondence: carlosgrande@mail.uniatlantico.edu.co; Tel.: +57-5-3852266

Table S1. Measurements of fiber diameters in scaffolding

	Diameters (nm)		
**Fiber Number**	0 % GO	0.5% GO	1% GO
1	128.06	169.71	272.03
2	101.98	128.06	144.22
3	161.25	107.7	226.27
4	126.49	161.25	252.98
5	134.16	172.05	160
6	161.25	178.89	161.25
7	197.99		
Mean	**144.45**	**152.94**	**202.79**
Desv. stand	**31.44**	**28.48**	**54.50**
